# Association between obesity and depressive symptoms in Mexican population

**DOI:** 10.1007/s00127-018-1517-y

**Published:** 2018-04-19

**Authors:** Gerardo A. Zavala, Spyros Kolovos, Alessandro Chiarotto, Judith E. Bosmans, Maiza Campos-Ponce, Jorge L. Rosado, Olga P. Garcia

**Affiliations:** 1 Faculty of Science, VU Amsterdam University, De Boelelaan 1091, 1081 HV Amsterdam, The Netherlands; 2 School of Natural Sciences, Autonomous University of Queretaro, Avenida de las Ciencias s/n, 76230 Querétaro, México

**Keywords:** Depression, Obesity, ENSANUT 2012, Mexico

## Abstract

**Purpose:**

Obesity and depression are among the leading causes of disability in Mexico, but their association has not been explored yet. The aim of the current study was to investigate the association between obesity and depression in Mexican population.

**Methods:**

We used data from the health and nutrition survey (ENSANUT 2012), which is representative of the Mexican population. Obesity was determined using the body mass index (BMI) and abdominal obesity by measuring waist circumference. Depressive symptoms were reported using the Center for Epidemiological Studies Depression Scale Short-Form (CES-D-SF, scale 0–21). Regression analyses were performed between obesity and depression, adjusting for gender, age, living with a partner, education, and diabetes history.

**Results:**

Obese women had 1.28 (95% CI 1.07–1.53) times the odds of having depression in comparison with normal-weight women, whereas no association was found for men (OR 0.94; 95% CI 0.74–1.19). A significant association between BMI and depressive symptoms score (*β* = 0.05, 95% CI 0.02–0.07) was present in women, but no association was found for men (*β* = − 0.02, 95% CI − 0.05 to 0.00). There was a statistically significant association between waist circumference and depression scores again for women (*β* = 0.03, 95% CI 0.01–0.04) but not for men (*β* = 0.00, 95% CI − 0.01 to 0.01). No associations were found between abdominal obesity and depression for both genders. No association was found between different obesity severity levels and depression for both genders.

**Conclusion:**

Obesity was associated with depression in Mexican women, whereas no association was found between obesity and depression in men.

## Introduction

Depression is currently among the leading causes of disability worldwide [1, 2] and it is related to large societal costs due to the increased healthcare utilization and productivity losses [3]. Furthermore, depression is associated with deterioration in health-related quality-of-life (QoL) and impairments in mental, physical, and social functioning of the individuals [4]. In accordance with global prevalence rates, depression is very common in Mexico with a 12-month prevalence of 6.1% and a lifetime prevalence of 12.8% [5, 6]. The impact of depression on the Mexican population has increased by 14% between 1990 and 2013, in terms of Years Lived with Disabilities (YLDS), and depression is ranked among the ten leading causes of disability in the country [7]. A prevalent condition that often occurs in conjunction with depression is obesity [8].

Obesity is a risk factor for a broad range of chronic diseases, such as cardiovascular disorders and type II diabetes [9]. It has been associated with impaired QoL and decreased life expectancy [10]. In addition to the increased costs due to the healthcare utilization, obesity has been associated with high unemployment rates, absenteeism, and productivity losses, and thus, it constitutes a large societal economic burden [11]. Mexico is the country with one of the highest combined prevalence of overweight and obesity (70%) in the world, according to the Health and Nutrition survey 2012 (ENSANUT 2012) [12].

It has been hypothesized that obesity and depression are related [8] and some authors describe the comorbidity between obesity and depression as an epidemic [13]. The previous studies have shown conflicting findings, with some indicating that obesity is related to higher depressive symptoms [14], others found no association between obesity and depression [15], and others found that obesity was related to lower depressive symptoms [16]. A meta-analysis synthesizing the results from the previous community-based studies found a modest association (pooled odds ratio = 1.18) between obesity and depression [17]. Most of the studies identified a stronger relationship between obesity and depression in women, and in individuals with lower educational level [17, 18]. Other studies have demonstrated that this relationship changes according to ethnicities and races [19, 20]. Moreover, it has been proposed that the association between obesity and depression is dependent on the measures of obesity used [e.g., body mass index (BMI) categories, or abdominal obesity] [21]. Even though Mexico is a country with one of the highest combined rates of overweight and obesity (> 70%), there are no studies evaluating the association between obesity and depression. Furthermore, evidence is missing in the context of Mexico regarding different measures of obesity (i.e., BMI, waist circumference, severity of obesity, and abdominal obesity) and their association with depression.

Considering these premises, this study investigated the association between obesity and depression in the Mexican population, using data representative of this population. More specifically, this study examined: (1) whether different BMI categories were associated with depression; (2) whether abdominal obesity was associated with depression; (3) whether the severity of obesity (i.e., mild, moderate, and severe obesity) was related to depression; (4) whether BMI and waist circumference measured as continuous variables were associated with continuous depressive symptom severity scores. The latter research question aimed to examine the robustness of the results using a different operationalization of obesity.

## Methods

### Study population

We used public data available from the Mexican National Health and Nutrition Survey (ENSANUT) [12]. This probabilistic survey is representative for the Mexican population at national, state and municipality level, and includes urban and rural strata. Data collection was conducted between October 2011 and May 2012, and the methodology is described in detail elsewhere [12]. The survey was administered to 57,097 households. Households where no one was available were excluded from the survey (13%) resulting in 50,528 available households. All participants signed an informed consent before answering questions and the survey was approved by the ethics committee of the National Institute of Public Health [22]. Individuals were included if they were at least 20 years old. The present study was reported according to the STROBE Statement [23].

### Depression

Depressive symptom severity was measured by the Spanish version of the Center for Epidemiological Studies Depression Scale Short-Form (CESD-SF), which has been previously used in the Mexican population [24]. This scale is a self-report unidimensional and reliable questionnaire used to evaluate depression severity over the last 2 weeks [25]. The CESD-SF consists of 7 items that can be scored on a 0–3 Likert scale, and giving a total score ranging from 0 to 21; higher scores indicate more severe depressive symptoms. A cut-off score ≥ 8 demonstrated satisfactory criterion validity with the original CES-D cutoff [25] and was used in this study to categorize participants as having at least mild depressive symptoms (reported as depression hereafter). Participants with a CESD-SF score of 7 or less were considered as having minor or no depressive symptoms.

### Anthropometric measurements

To calculate BMI, weight and height were measured by trained personnel [22]. Height was measured with a precision of 0.1 cm using a stadiometer. Weight was measured using a calibrated digital scale, with a precision of 1 g. Waist circumference was measured to the nearest 0.1 cm at the minimum circumference between the bottom of the ribs and the top of the iliac using a flexible fiber glass anthropometric tape [22]. The rates of underweight (BMI < 18.49), normal weight (BMI = 18.5–24.9), overweight (BMI = 25–29.9), and obesity (BMI > 30) were calculated according to the World Health Organization (WHO) cut-off points [26]. Obesity was further categorized in three severity levels, namely, obesity I (BMI = 30–34.9), obesity II (BMI = 35–39.9), and obesity III (BMI ≥ 40) [26].

Abdominal obesity was defined using the cut-off points from the Health Ministry of Mexico; male participants with waist circumference above 90 cm and female participants with a waist circumference above 80 cm were classified as abdominally obese [27].

### Statistical analysis

We used the “svy” command in Stata (version 13) to conduct all the analyses, adjusting estimates for the complex survey design while taking in consideration the expansion factor, the strata and primary sampling unit parameters to establish that the results are representative of the Mexican population. Clinical and demographic characteristics of the participants were reported using descriptive statistics.

To answer the first research question, we performed logistic regression analysis using the categorized BMI as independent variable (underweight, normal weight, overweight, and obesity, transformed into dummy variables) and depression as dependent variable with a dichotomous format (CES-D-SF score ≥ 8). For the second research question, we performed a logistic regression analysis, in which abdominal obesity was used as a dichotomous independent variable and depression was treated as a dichotomous-dependent variable. To examine the third question, a logistic regression analysis using three categories of obesity severity was treated as the independent variables and depression as the outcome variable. Finally, to answer the forth research question, we carried out two separate linear regression analyses: one with BMI as a continuous independent variable and one with waist circumference as a continuous independent variable and depressive symptom severity score as dependent variable using the continuous score; in this analysis, participants with a BMI lower than 18.5 (underweight) were excluded to assure a linear relationship between the independent variable and the dependent variable.

In all regression models, we included as potential confounders the variables gender, age, having a partner, history of diabetes, and education level stratified in three categories: ‘no education’, ‘middle’ (primary or secondary education), ‘high’ (more than secondary education), this categorization has been used previously by Villalpando et al. [28]. To determine whether there was confounding, we used the ‘rule of thumb’ of 10% change in the random coefficients between the model without covariates (crude model) and the model with covariates (adjusted model) [29]. The variables that were included as confounders were also checked as effect modifiers. Gender was an effect modifier, and therefore, all models were also stratified and presented according to gender. The regression coefficient (beta) or odds ratio (OR) was presented with their 95% confidence intervals (95% CI) and considered significant if the *p* value was < 0.05.

## Results

### Population characteristics

The demographic and clinical characteristics of the participants are summarized in Table 1. A total of 45,052 participants for whom a CESD-SF score was available were included in the analyses. The mean age was 41 (SD = 30.35) years and 53% of the participants were women. Fifty-five percent of the participants lived with a partner, 74% were living in urban areas, and 8% attained low education, 61% middle, and 31% high. One percent of the participants were underweight, 28% normal weight, 39% overweight, and 33% obese, whereas the mean BMI was 28.3 (SD = 10.83). From the participants with obesity, 89% had obesity I, 7% obesity II, and 4% obesity III. Abdominal obesity was reported for 74% of the participants and the mean waist circumference was 93.56 cm (SD = 27.17). Ten percent of the participants reported previous diagnosis of diabetes and 11% previous diagnosis of depression. The mean CES-D-SF score was 3.78 (SD = 10.83). A total of 7845 participants (16%) had a score of 8 or above on CES-D-SF (Table 1).

**Table 1:** Characteristics of the study population

Characteristic	Total (*n* = 45,052)	Depression (*n* = 7845; 16%)	No depression (*n* = 37,207; 84%)
Female, %	53	70	48
Age, mean (SD)	41 (30.35)	45 (28.49)	41 (30.47)
Married, %	55	52	57
Rural area, %	26	26	24
Education, %			
Non	8	11	6
Middle	61	67	60
High	31	22	34
Socioeconomic status, %			
Low	27	33	26
Middle	33	34	32
High	40	33	42
Indigenous language (yes)	9.3	9.3	10.4
BMI, mean (SD)	28.3 (10.83)	28.95 (10.41)	28.16 (10.50)
BMI categories, %			
Underweight	1	1	1
Normal weight	28	25	28
Overweight	39	35	40
Obesity^a^	33	39	32
Abdominal obesity, mean (SD)	74 (0.85)	80	73
Waist circumference, mean (SD)	93.56 (27.17)	94.57 (25.25)	93.35 (26.34)
CES-D-SF, mean (SD)	3.78 (10.83)	12.46 (7.52)	1.87 (4.47)
Previous diabetes diagnosis, %	10	14	8
Previous depression diagnosis, %	11	25	8

*BMI* body mass index, *CES-D-SF* Center for Epidemiological Studies Depression Short-Form

^a^Severity of obesity: obesity I = 89%; obesity II = 7%; obesity III = 4%

### Obesity and depression

#### BMI categories

Table 2 shows the crude and adjusted logistic regression models examining the association between BMI categories and depression. In the adjusted model, obese women had 1.28 (95% CI 1.07–1.53) times the odds of having depression in comparison with normal-weight women and this association was statistically significant (Table 2). There was no statistically significant association between obesity and depression for men (OR 0.94, 95% CI 0.74–1.19). For both men and women, there was no association between underweight or overweight BMI categories and depression (Table 2).

**Table 2:** Association between BMI categories and depression

	Overall				Female				Male			
	OR	95% CI		*p*	OR	95% CI		*p*	OR	95% CI		*p*
Normal weight^a^	1.00	–	–	–	1.00	–	–	–	1.00	–	–	–
Underweight^a^	1.00	0.68	1.49	0.99	0.87	0.55	1.38	0.56	1.13	0.53	2.41	0.76
Overweight^a^	0.97	0.88	1.09	0.65	1.03	0.90	1.19	0.64	0.88	0.72	1.08	0.23
Obese^a^	1.37	1.23	1.53	< 0.01	1.38	1.19	1.60	< 0.01	0.99	0.79	1.24	0.92
Normal weight	1.00	–	–	–	1.00	–	–	–	1.00	–	–	–
Underweight	0.83	0.43	1.60	0.58	0.65	0.33	1.29	0.22	1.72	0.51	5.80	0.38
Overweight	0.86	0.74	0.98	0.03	0.89	0.74	1.07	0.21	0.81	0.64	1.02	0.07
Obese	1.16	1.01	1.33	0.04	1.28	1.07	1.53	0.01	0.94	0.74	1.19	0.60
Gender (female)	2.29	2.03	2.59	< 0.01	–	–	–	–	–	–	–	–
Age (years)	1.01	1.01	1.01	< 0.01	1.01	1.01	1.02	< 0.01	1.00	1.00	1.01	0.24
Diabetes (yes)	1.52	1.29	1.78	< 0.01	1.28	1.03	1.58	0.02	2.04	1.56	2.65	< 0.01
Education (no)	2.20	1.76	2.75	< 0.01	1.88	1.43	2.47	< 0.01	3.05	2.11	4.43	< 0.01
Education (medium)	1.58	1.36	1.84	< 0.01	1.41	1.17	1.71	< 0.01	1.97	1.53	2.54	< 0.01
Partner (no)	1.11	0.91	1.35	0.31	1.09	0.89	1.33	0.39	1.23	0.70	2.19	0.47

*OR* odds ratio, *95% CI* 95% confidence interval

^a^Crude model

#### Abdominal obesity

Table 3 shows the results of crude and adjusted logistic regression models examining the association between abdominal obesity and depression. In the adjusted model, abdominal obesity was not associated with depression neither for men (OR 0.89, 95% CI 0.72–1.09) nor for women (OR 1.18, 95% CI 0.9–1.47) (Table 3).

**Table 3:** Association between abdominal obesity and depression

	Overall				Female				Male			
	OR	95% CI		*p*	OR	95% CI		*p*	OR	95% CI		*p*
Abdominal obesity^a^	1.44	1.29	1.60	< 0.01	1.34	1.15	1.55	< 0.01	1.01	0.85	1.20	0.92
Abdominal obesity	1.02	0.88	1.18	0.83	1.18	0.95	1.47	0.13	0.89	0.72	1.09	0.25
Gender (female)	2.39	2.11	2.69	< 0.01	–	–	–	–	–	–	–	–
Age (years)	1.01	1.00	1.01	< 0.01	1.01	1.01	1.02	< 0.01	1.00	1.00	1.01	0.19
Diabetes (yes)	1.55	1.32	1.81	< 0.01	1.30	1.05	1.60	0.02	2.05	1.57	2.68	< 0.01
Education (no)	2.26	1.80	2.82	< 0.01	1.93	1.46	2.55	< 0.01	3.06	2.10	4.47	< 0.01
Education (medium)	1.63	1.39	1.90	< 0.01	1.47	1.21	1.78	< 0.01	1.95	1.51	2.52	< 0.01
Partner (no)	1.12	0.91	1.38	0.27	1.10	0.89	1.35	0.37	1.29	0.73	2.28	0.39

*OR* odds ratio, *95% CI* 95% confidence interval

^a^Crude model

#### Severity of obesity

Table 4 presents the results of crude and adjusted logistic regression models examining the association between different obesity severity categories and depression. The results of the adjusted models showed no statistically significant association between the categories for severity of obesity and depression for men or women (e.g., for severe obesity OR 1.00, 95% CI 0.50–2.01 and OR 0.90, 95% CI 0.62–1.30 for men and women, respectively) (Table 4).

**Table 4:** Association between severity of obesity categories and depression

	Overall				Female				Male			
	OR	95% CI		*p*	OR	95% CI		*p*	OR	95% CI		*p*
Obese I (BMI 30–35)^a^	1.00	–	–	–	1.00	–	–	–	1.00	–	–	–
Obese II (BMI 35–40)^a^	1.01	0.85	1.21	0.86	1.06	0.89	1.26	0.51	1.34	0.86	2.11	0.20
Obese III (BMI < 40)^a^	1.27	0.98	1.66	0.07	1.42	1.08	1.88	0.01	0.99	0.55	1.79	0.98
Obese I (BMI 30–35)	1.00	–	–	–	1.00	–	–	–	1.00	–	–	–
Obese II (BMI 35–40)	0.92	0.75	1.14	0.46	0.86	0.68	1.08	0.20	1.22	0.77	1.95	0.40
Obese III (BMI < 40)	0.90	0.65	1.25	0.54	0.90	0.62	1.30	0.57	1.00	0.50	2.01	1.00
Gender (female)	2.71	2.18	3.37	< 0.01	–	–	–	–	–	–	–	–
Age (years)	1.01	1.00	1.01	0.06	1.01	1.00	1.02	0.02	1.00	0.98	1.01	0.72
Diabetes (yes)	1.40	1.08	1.82	0.01	1.11	0.84	1.47	0.48	2.43	1.50	3.94	< 0.01
Education (low)	2.34	1.58	3.46	< 0.01	2.02	1.34	3.06	< 0.01	3.49	1.44	8.45	0.01
Education (medium)	1.65	1.29	2.11	< 0.01	1.45	1.08	1.94	0.01	2.41	1.52	3.81	< 0.01
Partner (no)	1.15	0.83	1.59	0.41	1.14	0.85	1.52	0.38	1.14	0.38	3.39	0.82

*OR* odds ratio, *95% CI* 95% confidence interval

^a^Crude model

#### BMI and waist circumference continuous

Table 5 presents the results of crude and adjusted linear regression models examining the association between BMI (continuous) and depressive symptom severity score, and waist circumference (continuous) and depressive symptom severity score. In the former adjusted model, there was a statistically significant association between BMI and depression scores for women (*β* = 0.05, 95% CI 0.02–0.07), meaning that when BMI increased by one depression score increased by 0.05. In the latter adjusted model, there was a statistically significant association between waist circumference and depression scores again for women (*β* = 0.03, 95% CI 0.01–0.04), meaning that when waist circumference increased by one depression score increased by 0.05. There were no statistically significant associations for men (Table 5).

**Table 5:** Association between BMI and waist circumference with depressive symptoms severity score (CES-D-SF)

	Overall				Female				Male			
	*β*	95% CI		*p*	*β*	95% CI		*p*	*β*	95% CI		*p*
BMI^a^	0.05	0.04	0.06	< 0.01	0.06	0.04	0.08	< 0.01	− 0.02	− 0.04	0.01	0.20
BMI	0.02	0.00	0.04	0.03	0.05	0.02	0.07	< 0.01	− 0.02	− 0.05	0.00	0.08
Gender (female)	1.62	1.43	1.82	< 0.01	–	–	–	–	–	–	–	–
Age (years)	0.02	0.01	0.02	< 0.01	0.03	0.02	0.04	< 0.01	0.01	0.00	0.02	0.12
Diabetes (yes)	1.06	0.68	1.44	< 0.01	0.76	0.15	1.38	0.02	1.37	0.82	1.92	< 0.01
Education (no)	1.49	1.03	1.96	< 0.01	1.20	0.59	1.81	< 0.01	1.75	1.14	2.37	< 0.01
Education (medium)	0.92	0.71	1.13	< 0.01	0.85	0.52	1.19	< 0.01	0.93	0.70	1.16	< 0.01
Partner (no)	0.19	− 0.24	0.62	0.39	0.20	− 0.25	0.66	0.38	0.26	− 0.79	1.31	0.63

Waist circumference^a^	0.01	0.01	0.02	< 0.01	0.03	0.02	0.04	< 0.00	0.00	− 0.01	0.01	0.73
Waist circumference	0.01	0.00	0.02	< 0.00	0.03	0.01	0.04	< 0.00	0.00	0.99	1.00	0.36
Gender (female)	1.70	1.50	1.89	< 0.00	–	–	–	–	–	–	–	–
Age (years)	0.02	0.01	0.02	< 0.00	0.02	0.01	0.03	< 0.00	0.01	− 0.00	0.02	0.06
Diabetes (yes)	1.06	0.68	1.44	< 0.00	0.77	0.16	1.39	0.01	1.36	0.81	1.91	< 0.00
Education (no)	1.53	1.08	1.98	< 0.00	1.25	0.63	1.88	< 0.00	1.74	1.13	2.35	< 0.00
Education (medium)	0.97	1.39	1.19	< 0.00	0.92	0.58	1.26	< 0.00	0.97	0.70	1.17	< 0.00
Partner (no)	0.16	− 0.28	0.61	0.48	0.13	− 0.34	0.59	0.59	0.40	− 0.66	1.47	0.46

^a^ Crude model

## Discussion

The present study examined the association between obesity and depression in the Mexican population. We found that women categorized as obese using BMI cutoffs were more likely to experience depressive symptoms than women with normal weight. In addition, higher BMI and waist circumference were significantly related to increased depressive symptom severity for women, but these associations were not strong and may not be clinically relevant. We found no association between obesity and depression for men. In addition, abdominal obesity was not related to depression in both genders. Finally, there was no difference between different categories of severity of obesity and their association with depression.

Our findings are in line with the previous studies that found modest association between obesity and depression for women but not for men [17, 30–32]. In the Mexican context, gender-related differences in the association between obesity and depressive symptoms may be explained by cultural, ethnic, and social factors [33, 34]. Women are more exposed to media, such as magazines and TV, commonly showing “ideal bodies” in their programs and advertisements. The previous studies have shown that exposure to media showing attractive models or “ideal bodies” was related to lower body satisfaction [35]. In addition, women are more aware of female attractiveness as presented in the media than men, increasing their weight-related concern [36]. Furthermore, it appears that women experience more sociocultural pressure and stigma towards being thinner as compared to men increasing their susceptibility to depression [36, 37]. However, other biological, genetic, or environmental determinants, which could not be examined by the current study, might be also playing a role in the complex association between obesity and depression [8].

We found an association between obesity and depression when BMI categories were used, whereas no relationship was found when waist circumference was used as predictor. This indicates that the relationship between obesity and depression is influenced by the measure used to operationalize obesity. Waist circumference is a good proxy to evaluate risk of diseases such as cardiovascular risk or metabolic syndrome [38], but it is not an adequate reflection of the construct obesity especially for women for whom gynecoid obesity (i.e., largest accumulation of fat around the hips) is common [39].

We found a statistically significant association between BMI and waist circumference (as continuous variables) with depression for women. However, the beta coefficients were very small and the association may not be clinically relevant. Similar to our findings, different studies have found no association or a marginal association between BMI (continuous) with depressive symptoms [40] and no association between waist circumference and depressive symptoms [14]. Furthermore, various clinical and demographic characteristics of the participants were included as covariates in the models. In most of the analyses, lower education and history of diabetes were associated with an increased risk for depression. The protective effect of higher education against depression has been found in the previous studies too [41]. Similarly, there is strong evidence in the literature showing the link between diabetes and depression [42]. Therefore, it is important that these participants’ characteristics are included as covariates when examining the relationship between obesity and depression.

### Strengths and limitations

One of the study’s strengths is the inclusion of a large sample size that is representative of the Mexican population. Moreover, the measurement of obesity was carried out by trained personnel using objective measures and did not rely on self-reporting. Finally, the study investigated two highly prevalent conditions in the Mexican population that are very relevant considering their enormous burden for society.

This study is not without limitations. First, the presence of depression was operationalized using a self-report scale. Therefore, the individuals were not formally diagnosed with depression, for which a structured clinical interview should be used. However, the CES-D-SF has good psychometric properties and is widely used to assess depressive symptom severity in the literature. Second, the study has a cross-sectional design that does not allow to draw any conclusions on the direction of causality between obesity and depression; future longitudinal studies can be used to infer a causal relationship. In addition, only current depressive symptoms were measured, and not the chronic course of depression, this is a limitation, since depressive symptom severity is known to fluctuate in longer periods (e.g., due to natural remission). Finally, the influence of other variables, such as psychiatric comorbidity, was not measured in the survey, and therefore, they were not included in the models.

### Implications

Given the high rates of obesity and depressive symptoms, future research should examine the casual pathways between obesity and depression in Mexico as well as the possibility of screening for depression in obese women [43, 44]. Furthermore, raising awareness among clinicians that there is an association between obesity and depression is crucial and it may assist in developing targeted preventive strategies for both conditions [45]. Finally, the effectiveness and cost-effectiveness of multidisciplinary lifestyle and psychological interventions for obese women with emotional complaints and psychological distress should be investigated.

## Conclusion

Obesity was modestly associated with depression in Mexican women. This association was statistically significant only when BMI categories were used. However, no relationship was found between obesity and depression for men.
